# Case Report: Acute myocarditis and cerebral infarction following *Bothrops lanceolatus* envenomation in Martinique: a case series

**DOI:** 10.3389/fcvm.2024.1421911

**Published:** 2024-11-28

**Authors:** Jonathan Florentin, Karim Farid, Hatem Kallel, Remi Neviere, Dabor Resiere

**Affiliations:** ^1^ Department of Critical Care Medicine, Toxicology and Emergency, CHU Martinique (University Hospital of Martinique), Fort-de-France, France; ^2^ Cardiovascular Research Team UR5_3 PC2E Pathologie Cardiaque, Toxicité Environnementale et Envenimations, Université des Antilles, Fort-de-France, France; ^3^ Institut Caribéen D’imagerie Nucléaire, Pole Imagerie Médicale CHU Martinique (University Hospital of Martinique), Fort-de-France, France; ^4^ Intensive Care Unit, Cayenne General Hospital, Cayenne, French Guiana

**Keywords:** *Bothrops lanceolatus*, snakebites, myocarditis, ischemic stroke, cardiovascular complications

## Abstract

*Bothrops lanceolatus* (Bl), a snake endemic to Martinique, is responsible for numerous envenomations annually, leading to severe complications such as thrombosis, necrosis, and hemorrhage. This case series investigates the link between *Bothrops lanceolatus* envenomation and acute myocarditis, utilizing cardiac magnetic resonance imaging (MRI) to enhance management strategies. In both cases, cardiac MRI confirmed myocarditis with edema, and subsequent cerebral MRI revealed bilateral infarcts. Elevated troponin levels further supported the myocarditis diagnosis. Multiple doses of Bothrofav® antivenom facilitated recovery without clinical after effects.These cases represent the first documented instances of myocarditis due to Bl envenomation confirmed via cardiac MRI. Prompt administration of antivenom and the use of advanced imaging techniques were crucial in achieving favorable outcomes.

## 1 Introduction

The *Bothrops lanceolatus* (Bl) snake, endemic to the Island of Martinique and a part of the Crotalinae family, is responsible for approximately 30 envenomation incidents annually. *Bothrops sp.* venom contains digestive enzymes and spreading factors causing local and systemic injuries. Key enzymes include hemorrhagic metalloproteinases (“hemorrhagins”) and phospholipase A2, responsible for edema, myotoxicity, and anticoagulant effects. Bl venom, with a uniqcue prothrombotic profile, includes phospholipases A2, serine proteinases, L-amino acid oxidases, metalloproteinases, and a specific C-type lectin-like molecule. Envenomation leads to pain, swelling, erythema, and potential severe complications like local necrosis and systemic thrombotic events. Severe cases show rapid swelling and systemic signs such as hypotension, tachycardia, and respiratory difficulties, even with initially moderate envenomation (1). The repercussions of its bite, if left untreated, are severe; while thrombosis is a common outcome, necrosis or hemorrhage can also occur, albeit less frequently (2, 3). The primary intervention involves specific serotherapy (1, 4, 5). In this article, we present two distinct cases of severe envenomation, both exhibiting symptoms indicative of myocarditis accompanied by thrombotic episodes. Despite many countries facing challenges with snakebites as a health concern and lacking resources like cardiac magnetic resonance imaging (MRI), our aim is to establish a link between *Bothrops lanceolatus* envenomation and acute myocarditis. We believe this revelation can potentially assist healthcare professionals managing viperine envenomations.

## 2 Clinical summary

### 2.1 Case 1

**Figure 1:**
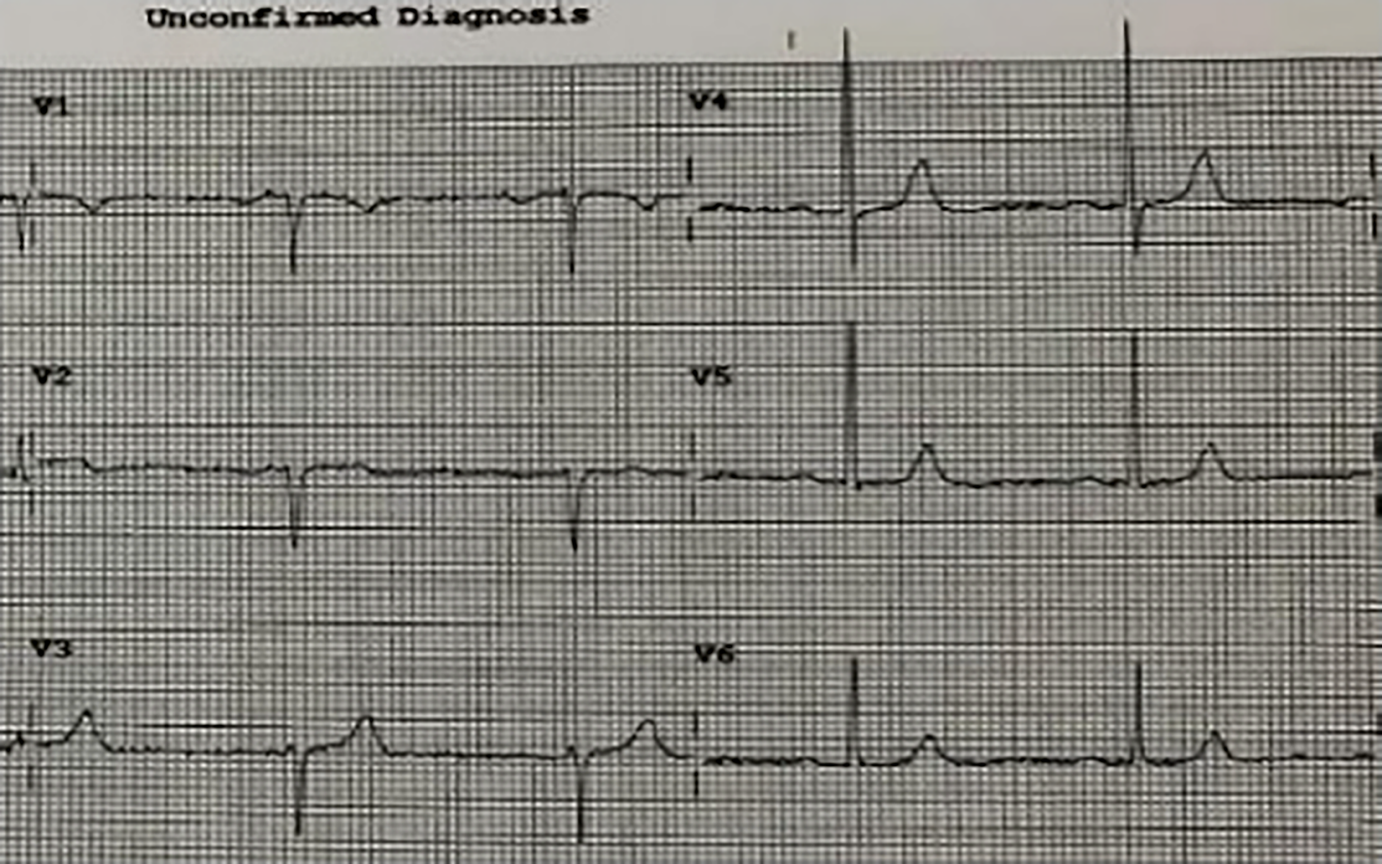
ECG H+1.5 after snake bite, patient no 1.

**Figure 2:**
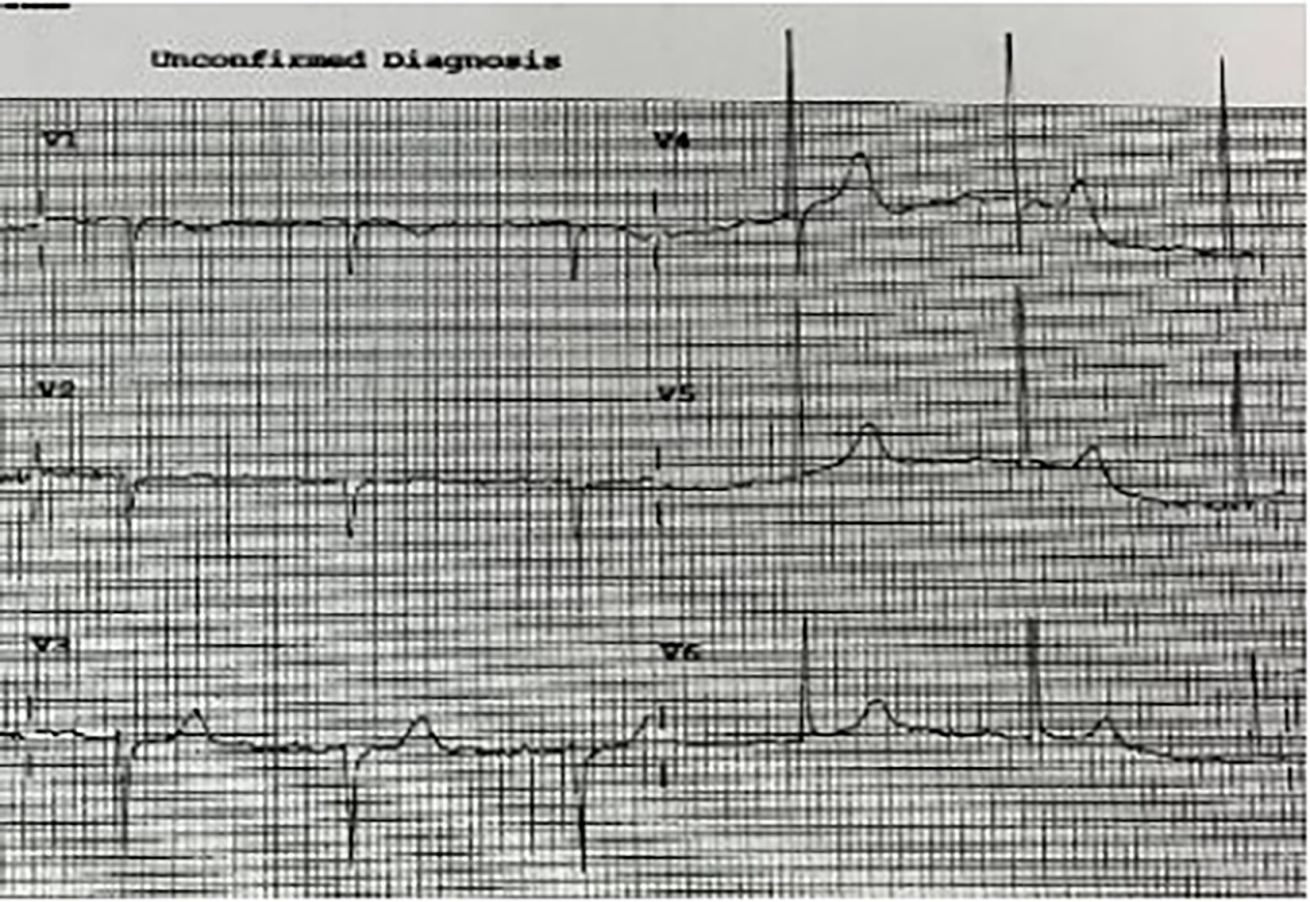
ECG H+27 showing widening and increased QRS amplitude, particularly at V3 and V4 in patient no. 1.

**Figure 3:**
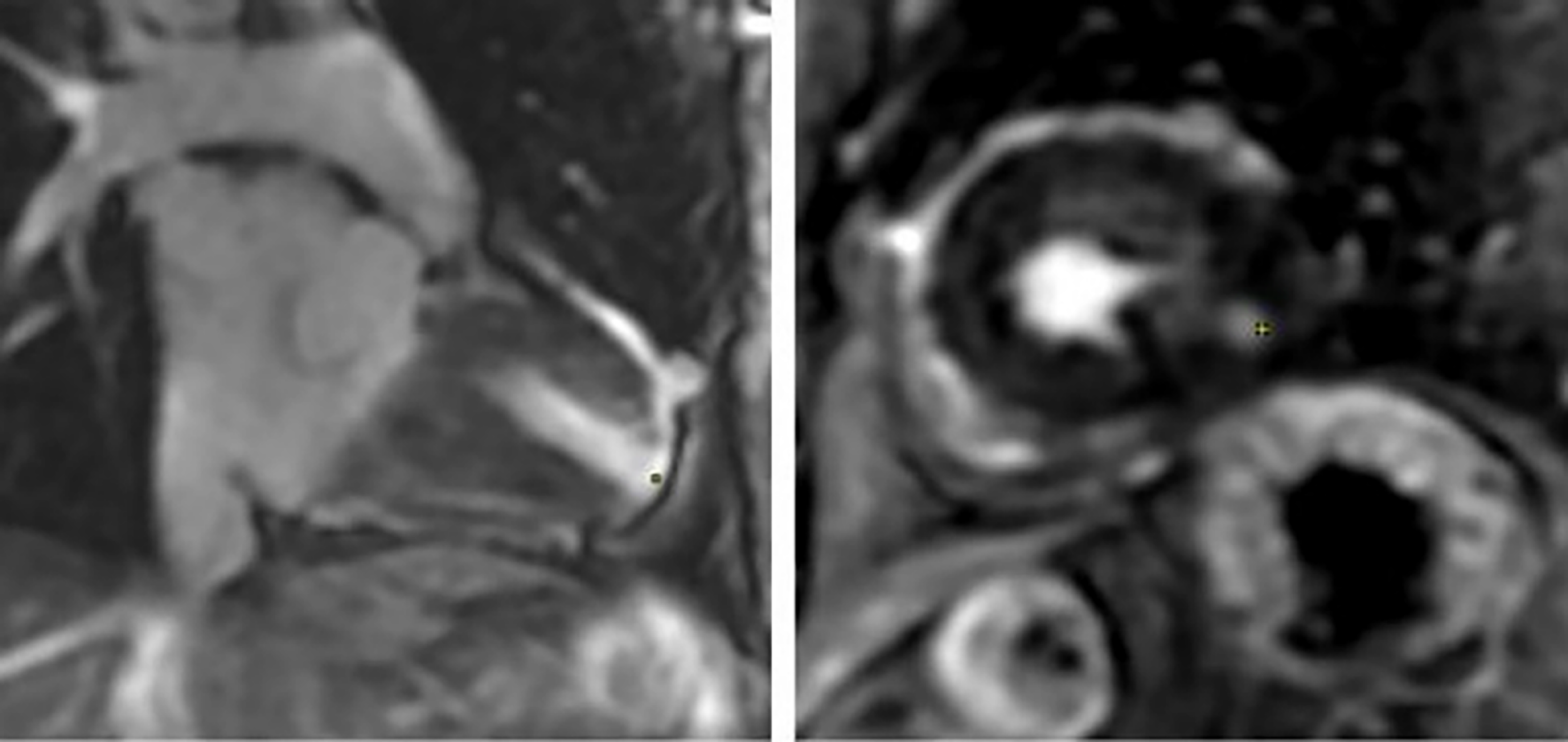
Coronary angiography from patient no.1 showing discreetly atheromatous coronaries.

**Figure 4:**
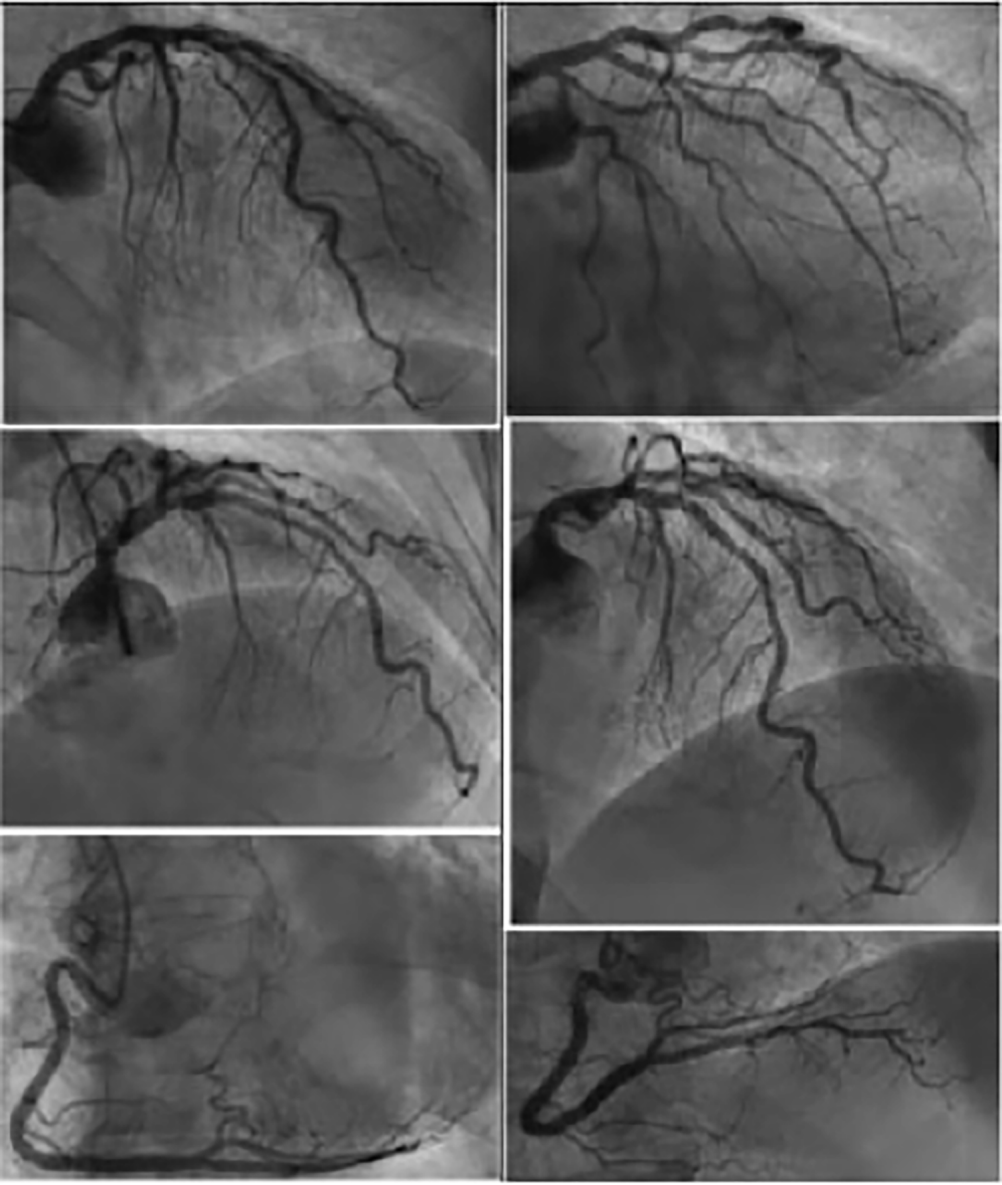
Cerebal MRI diffusion sequence from patient no.1 showing multiple supra- and infra-tentorial ischemic foci.

**Figure 5:**
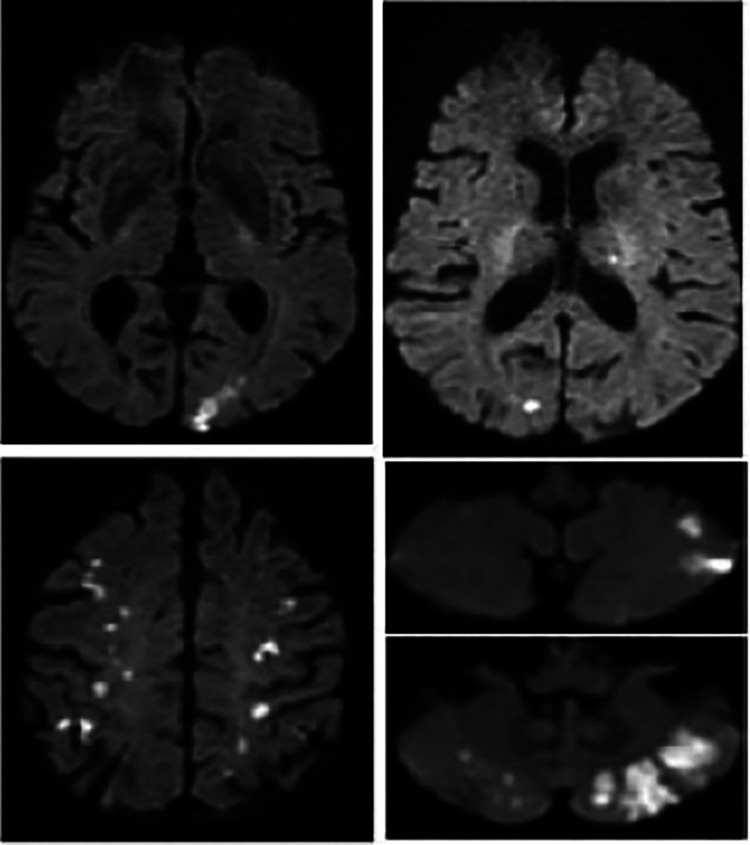
Multiparametric cardiac MRI from patient no. 1. Yellow spots showing late gadolinium enhancement at the apex as well as an anterior and lateral sub endocardiac and midwall enhancement.

A 93-year-old male, with a medical history of arterial hypertension and prostate cancer treated with amlodipine and ramipril, suffered a bite on his left hand from a juvenile Bl snake, approximately 37 cm in length. This occurred while he was tending to his garden on December 28, 2020, at 3:00 pm. An hour and a half later, upon his admission to the University Hospital of Martinique, he displayed inflammatory edema and pain on the back of his hand and wrist. Clear fang marks and palpitations were evident. His vital statistics were recorded as: blood pressure 172/74 mmHg and heart rate at 52 BPM. No neurological issues or headaches were detected. Furthermore, his initial electrocardiogram (ECG) appeared normal (Figure 1), first troponin-I level was inferior to 10 ng/ml. This set of symptoms aligned with a Grade 2 envenomation. Initial medical intervention involved a 10 ml intravenous administration of the specific anti-venom serum: Bothrofav 2®. However, within an hour of the treatment, the patient began experiencing epigastric chest pain. His blood pressure surged to 210/120 mmHg. The ECG registered an increase in the T-wave amplitude in the V4 derivation (Figure 2), and elevated troponin and Ddimers levels were noted (Table 1). Subsequent medical evaluations a pulmonary angioscan and cardiac echocardiogram did not reveal thrombosis or any abnormalities, the kinetics of the left ventricle and its ejection fraction were normal. Yet, by day 2, while his hand symptoms improved, his chest discomfort and rising troponin persisted. Alarming electrocardiogram readings prompted a cardiac MRI. The scan highlighted multiple new focal points suggestive of myocarditis, with edema evident on T2 (Figure 3). In parallel, a coronary angiogram ruled out coronary thrombus or significant stenosis (Figure 4). All these factors, combined with the absence of allergic or infectious symptoms, allowed us to rule out differential diagnoses such as Kounis syndrome. Additionally, a cerebral CT angiogram revealed the absence of cerebral infarction. Given the strong indications of acute myocarditis, a second 10 ml dose of the specific antivenom serum was administered 24 h post-bite. By day 4, while the hand wound displayed further healing and chest symptoms decreased, the patient reported a headache. A cerebral MRI then disclosed bilateral hemispheric and cerebellar infarcts, indicative of stroke from thrombotic macroangiopathy following a Bl bite (Figure 5). This progression to Grade 4 envenomation warranted a third 10 ml administration of the specific antivenom serum. Thankfully, the patient's condition subsequently improved, and he was discharged on January 4, 2021. Upon follow-up evaluations 10 days and 1-month post-envenomation, the patient displayed no neurological or cardiological aftereffects. He seamlessly resumed his routine activities, maintaining the same level of autonomy he had prior to the incident.

**Table 1:** Evolution of biological prognostic characteristics in patient no. 1.

Prognostic features	H + 1.5	H + 2	H + 5	H + 8	H + 14	H + 22	H + 24	H + 38	H + 62	H + 72	H + 86	H + 116	H + 134	H + 158
Troponine (pg/ml)	<10		55	400.6	3,327.4	11,374.6		11,292.8	12,993.6	9,000.5	7,915.3	4,097.7	2,059	1,104.3
CPK (UI/L)	114		125		407	694						118	90	64
PT (%)	101				101	100		96	99		97	100	101	104
Ddimere (µg/ml)	1.42													
Platelets (G/L)	205				201			191	166		161	177	188	203
Leukocytes (G/L)	7.04				10.05	10.47			9.31		8.4	7.71		
C-reactive protein (mg/ml)	0.4				3.6	7.7		14.4	15.6		11.5	4.8		
Dose of Bothrofav2 ® (10 ml)		1					1			1				

### 2.2 Case 2

**Figure 6:**
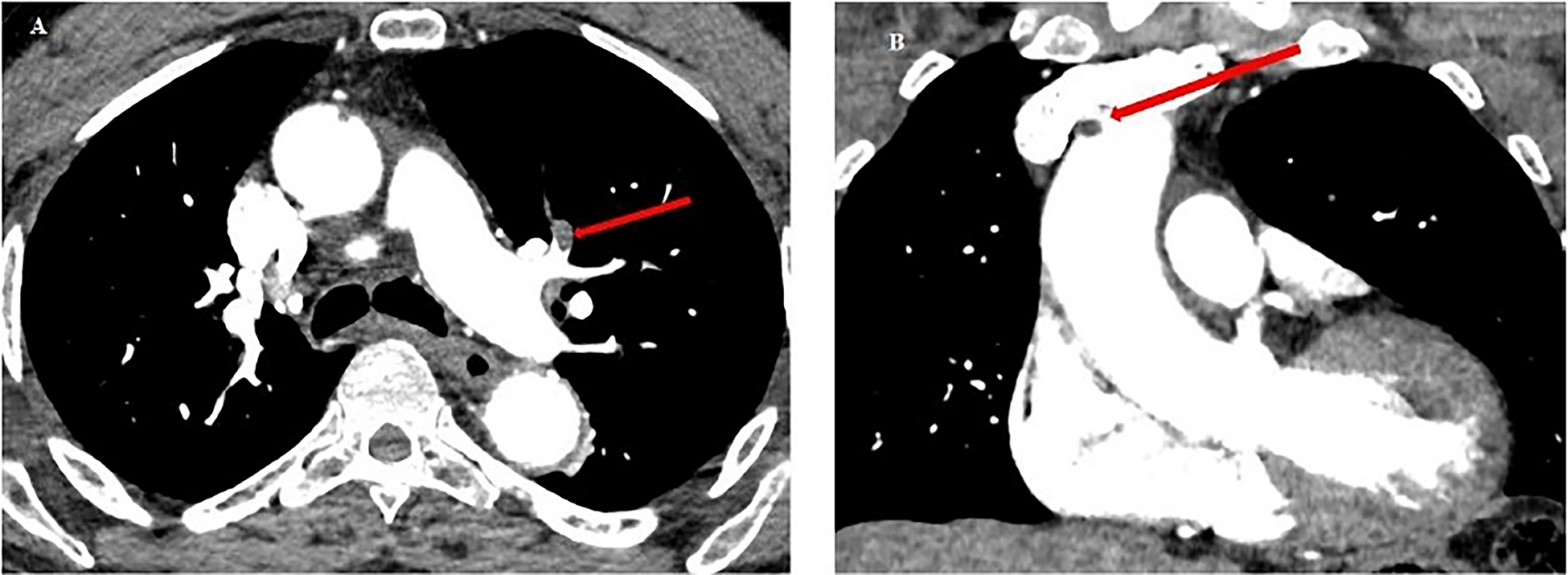
Chest CT scan from patient no.2. **(A)** Red arrow: pulmonary embolism of the upper lingular segment. **(B)** Red arrow: thrombus in the lumen of the ascending aorta (5 mm diameter), and of the aortic arch at the level of the birth of the brachiocephalic arterial trunk (6 mm).

An 84-year-old man with no significant medical history experienced envenomation on October 29, 2021, at 10:30 a.m. on his left foot while working in tall grass. The offending animal was not identified. Post-envenomation, the patient managed to walk but experienced lipothymic malaise three hours later, leading him to seek help at the fire station. Upon hospital admission 4.5 h post-bite, the clinical examination showed localized, non-extensive edema on the foot's dorsum, accompanied by moderate pain without bleeding. Vital statistics were recorded as: blood pressure at 120/79 mmHg and heart rate at 87 BPM. There were no apparent general neurological symptoms or chest discomfort, and his initial ECG was normal. Despite this, he was administered a 10 ml dose of Bothrofav® at the 7-h mark post-envenomation. However, initial blood tests showed elevated troponin levels at 160 pg/ml, a Ddimers level at 92.24 µg/ml, and a decreased PT at 66% (Table 2). With a subsequent rise in troponin to 333 pg/ml, a thoracic and cerebral CT scan was conducted 11.5 h post-bite (Figure 6). The scan disclosed pulmonary and aortic thrombosis without cerebral thrombosis, and a cardiac echocardiogram did not reveal abnormalities the kinetics of the left ventricle and its ejection fraction were normal.

**Table 2:** Evolution of biological prognostic characteristics of patient no. 2.

Prognostic characteristics	H + 5	H + 7	H + 10	H + 12	H + 15	H + 19	H + 28	H + 43	H + 67	H + 91	H + 115	H + 139
Troponine (pg/ml)	160		333			18,451	14,074	7,028.7	4,732.4	3,456.8	1,198.4	
CPK (UI/L)	192					854	723	384		137		
PT (%)	66					65	80	84	92	93	90	83
Ddimere (µg/ml)	92.24							7.24	5.44			
Platelets (G/L)	105					68		68	78	97	123	149
Leukocytes (G/L)	11.96					9.19	7.62	8.44	7.88	8.94	7.81	
C-reactive protein (mg/ml)	3.4					15.1	23.6	22.1	13.2	10	10.4	
Dose of Bothrofav2 ® (10 ml)		1		1	1							

**Figure 7:**
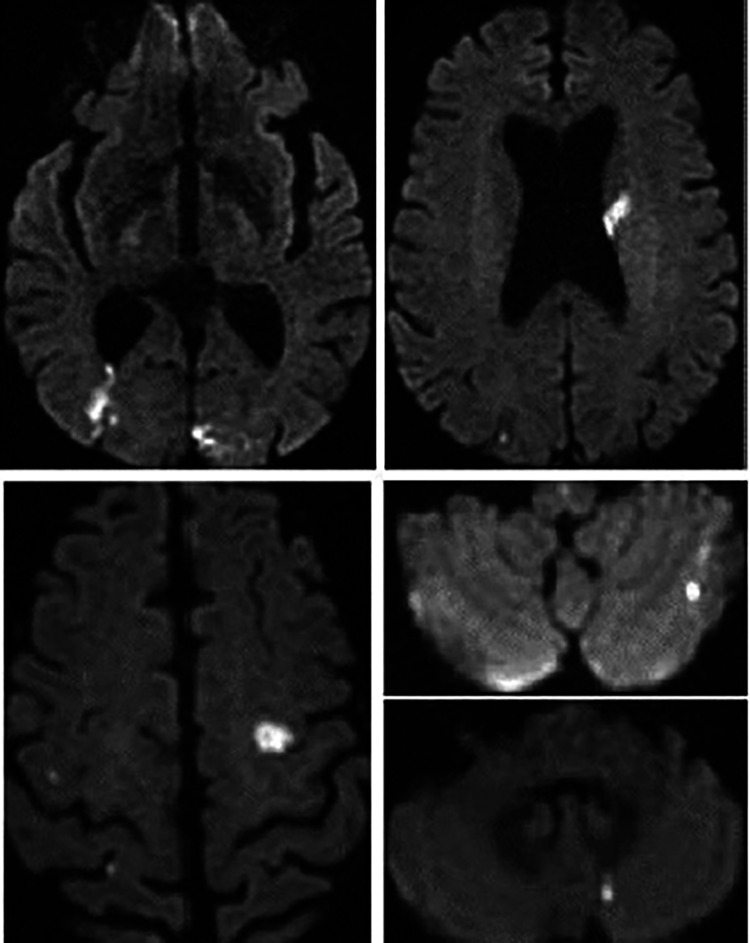
Patient no. 2 cardiac MRI: yellow spots showing Hypersignal latero basal and apico septal in T2 weighting, several patches of enhancement without coronary systematization frank: transmural latero basal and quasi transmural latero to the apex, as well as medio myocardial septal.

**Figure 8:**
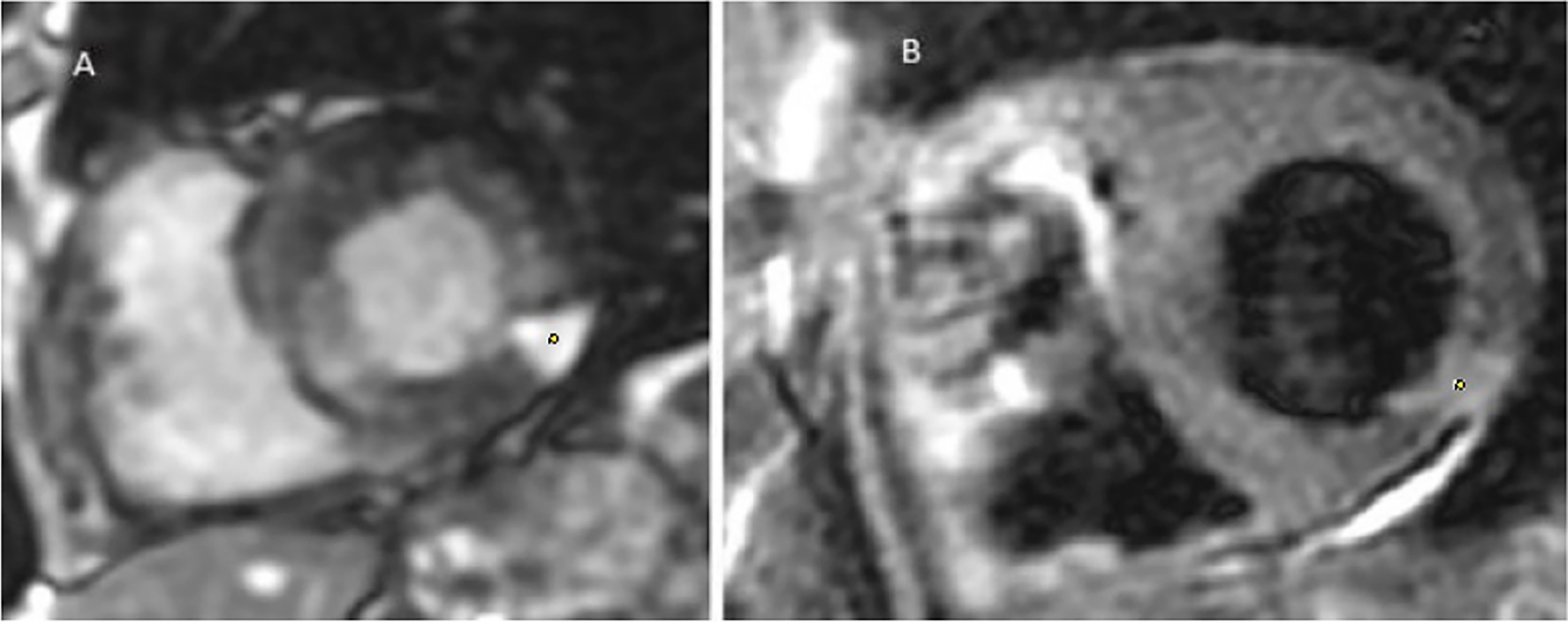
Cerebral MRI diffusion sequence in patient no. 2 showing multiple supra- and infra-tentorial ischemic foci.

In response to the imaging findings, the patient was given a second and third 10 ml dose of Bothrofav® at the 12-h and 15-h marks, respectively. After reporting a subjective sensation of a headache without any neurological deficit 22 h post-bite, a cerebral MRI was performed (Figure 7). The MRI detected multiple strokes, especially involving the PICA and junctional territories. Furthermore, due to the alarming rise in troponin to 18,451 pg/ml by the 19th hour, a cardiac MRI was carried out on Day 3. This MRI revealed a T2 hypersignal and late transmural lateral basal enhancement, indicative of a recent microvascular event. Multiple areas displayed late mediomyocardial or cocardial enhancement with fibrotic signs, suggesting cardiac amyloidosis (identified coincidentally) or myocarditis (Figure 8). However, unlike in the Patient No.1 case, the ECG did not exhibit any disruptions from the myocardial damage. After six days of hospitalization, the patient was discharged without any clinical aftereffects. He regained his physical activity level, matching his pre-envenomation state.

## 3 Discussion

Snakebite envenomations present as a pressing global public health challenge (6). While the cardiac toxicity of such envenomations has been observed, it remains insufficiently studied (7–9). Pathophysiological hypotheses postulate either a toxic effect of the venom directly on cardiac cells (10, 11) or a cardiac hypersensitivity to the toxins (12). Viperine envenomation-induced myocarditis is documented in animal studies (13, 14) but remains scarcely recorded in humans (10, 11, 15–17). Interestingly, myocarditis following a bothrops bite is yet to be reported. A pioneering instance where a cardiac MRI showcased myocarditis in a cobra bite context was in India, 2019 (17). The MRI indicated myocardial edema, evident as a T2-weighted hypersignal, with hypokinesis from basal to medial areas in the lateral wall. Numerous instances of ischemic accidents from Bl snakebites have been documented by Laurent Thomas (18) These incidents might be attributed to the effects of serine proteinase on the blood coagulation pathway. Additionally, P-III metalloproteinase is believed to facilitate prothrombin activation (19) and endothelium injuries. In the case of these patients, the known prothrombotic effect of Bl venom prompted us to conduct an extensive evaluation for thromboses, including thoracic CT scans and cerebral MRIs, in addition to cardiac examinations. To the best of our knowledge, this marks the first recorded case of myocarditis resulting from a Bl bite. “The application of cardiac MRI in the management of bothrops envenomations is unprecedented. Compared to the 2019 publication by Waitayangkoon & al, our two cases pinpointed microangiopathies on MRI (17). Anatomopathological examinations from a deceased patient post-Bl bite highlighted multifocal thrombotic microangiopathy (20). Both patients had unremarkable cardiac ultrasound results more specifically, showing the absence of ventricular kinetic disorders or changes in cardiac echogenicity suggestive of myocardial edema. Given the observations in patient no.1 and the absence of ECG anomalies, patient no.2 was not subjected to coronary angiography. For diagnosing myocarditis, cardiac MRI has emerged as an invaluable tool (12, 21, 22). Our experience emphasizes its superiority in specificity and sensitivity over cardiac echocardiography when identifying viperine envenomation-induced cardiac damage, a point echoed by Chara & al in 2017 (15). The progression in patient no.1, despite early treatment, is intricate. Most cases in literature suggest delayed, if any, antivenom treatment (11, 15). Yet, both Aravanis & al and Brown & al reported early antivenom administration (10, 16). The discord between local effects indicating grade 2 envenomation and subsequent cerebral infarction, characteristic of grade 4, in patient no.1 warrants exploration. Variabilities in venom composition or potency between juvenile and adult Bl snakes, as seen in *Bothrops atrox* and *Bothrops jararacussu* (23, 24), might play a role. Studies on Bl in this context are ongoing. The management protocol for envenomation, based on the early administration of a specific antivenom appropriate to the grade of envenomation, enabled a favorable outcome without sequelae for the patients (Table 3). While most countries grappling with snakebite as a public health issue do not have access to cardiac MRI, we aim to include BL envenomation in the known causes of acute myocarditis. We hope that this addition aids healthcare professionals treating patients with viperine envenomations.”

**Table 3:** Grading Bl and protocol for managing treatment with specific antivenom serum (1).

Grade severity		Symptoms	Dose of antivenom (Bothrofav 2 ®)
1	Minor	No swelling No pain No general signs	None
2	Moderate	Local swelling confined to 2 segments of the bitten limb Moderate pain No general signs	10 ml
3	Severe	Regional edema: extension of swelling beyond 2 segments Persistent and resistant pain to analgesics No general signs	20 ml
4	Major	Swelling spreading to the trunk General signs (vomiting, headache, abdominal or chest pain) Hypotension, thrombosis Isolated thrombocytopenia Disseminated intravascular coagulation	30 ml

## 4 Conclusion

These two cases mark the inaugural MRI documentation of acute myocarditis post-Bl bite. Swift specific treatment and cardiac MRI capabilities led to a favorable, sequela-free outcome. In these two cases, MRI surpasses injection-based CT in early brain lesion diagnosis and seemingly aligns with histological findings from cardiac tissues of the deceased. MRI stands out as the gold standard for diagnosing cardiac and neurological injuries from Bl bite envenomation. Although these two cases do not establish a guideline, the use of MRI in diagnosing cardiovascular complications of snakebite envenomation appears to be a promising solution for early severity assessment, especially when there is a discrepancy between clinical and paraclinical findings. However, further research is essential to discern potential variations in venom properties between juvenile and adult Bl snakes.

## Data Availability

The raw data supporting the conclusions of this article will be made available by the authors, without undue reservation.
